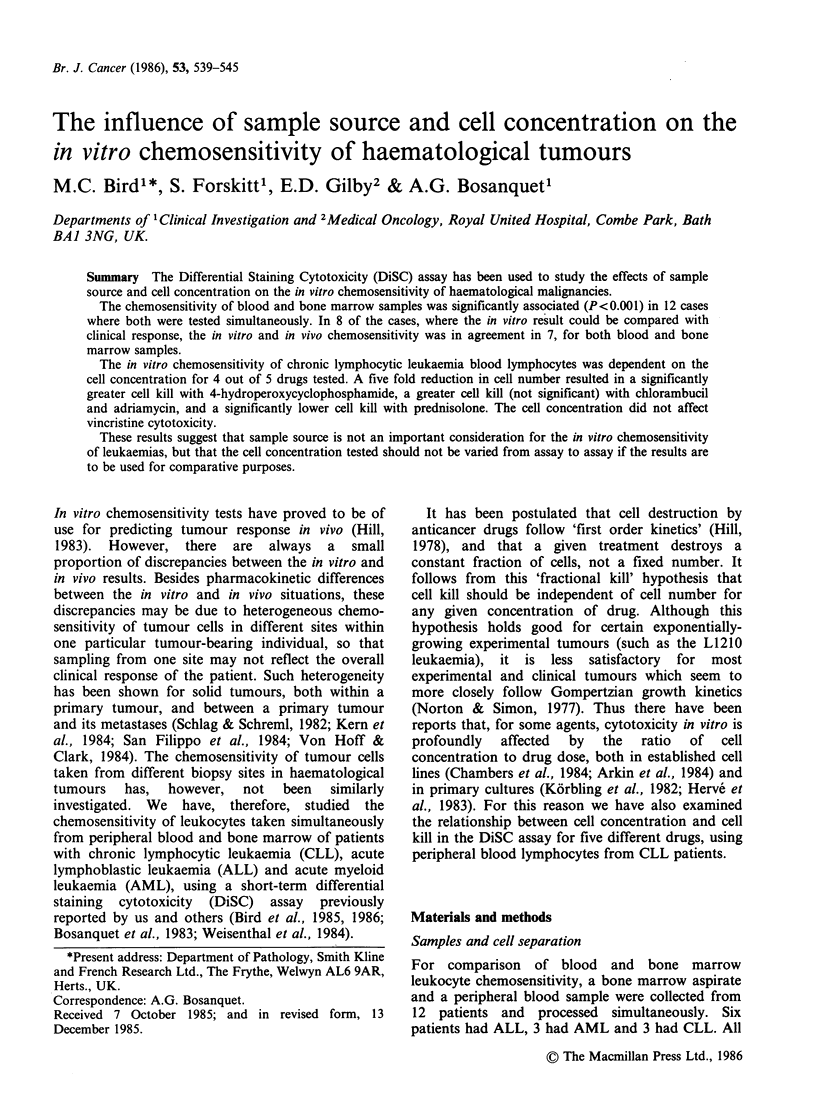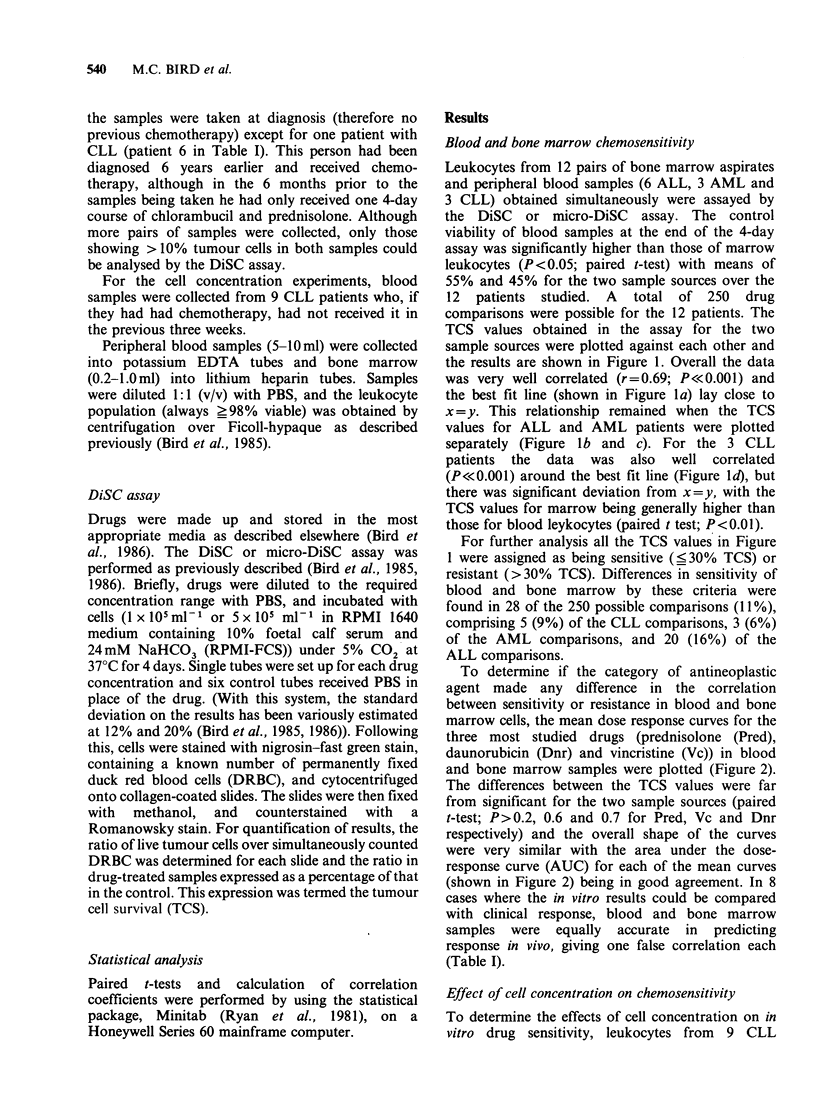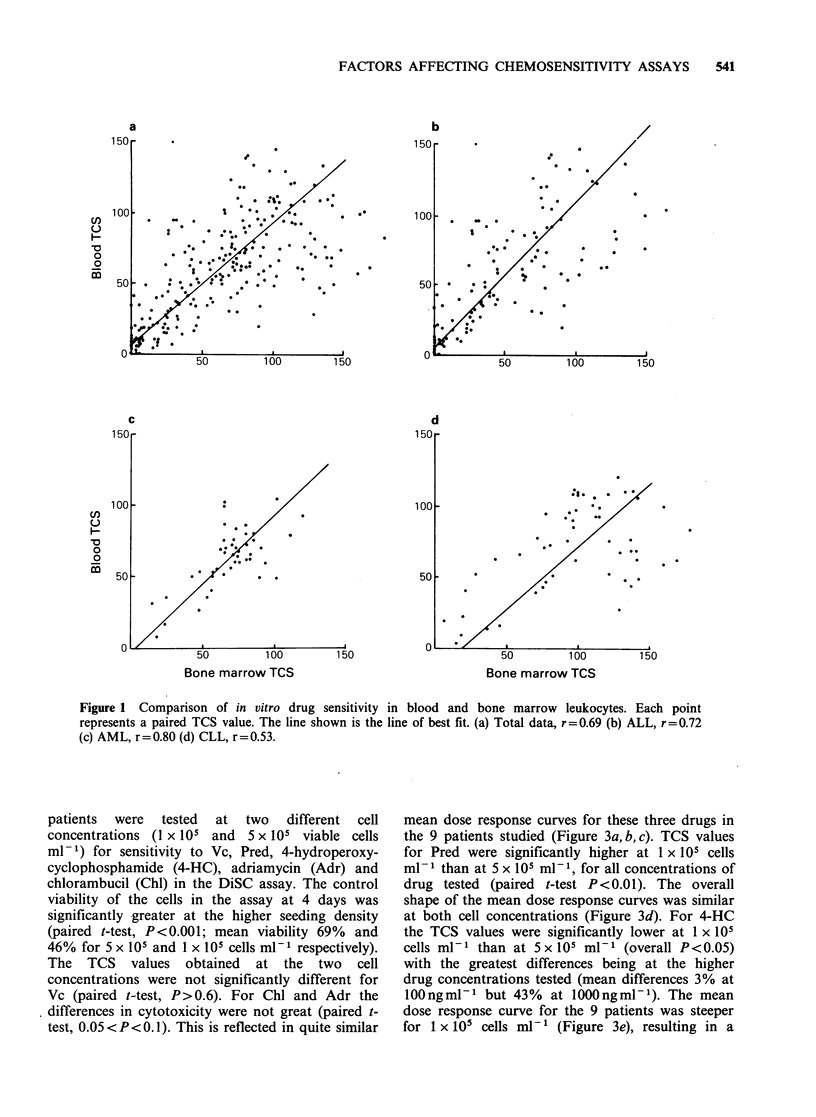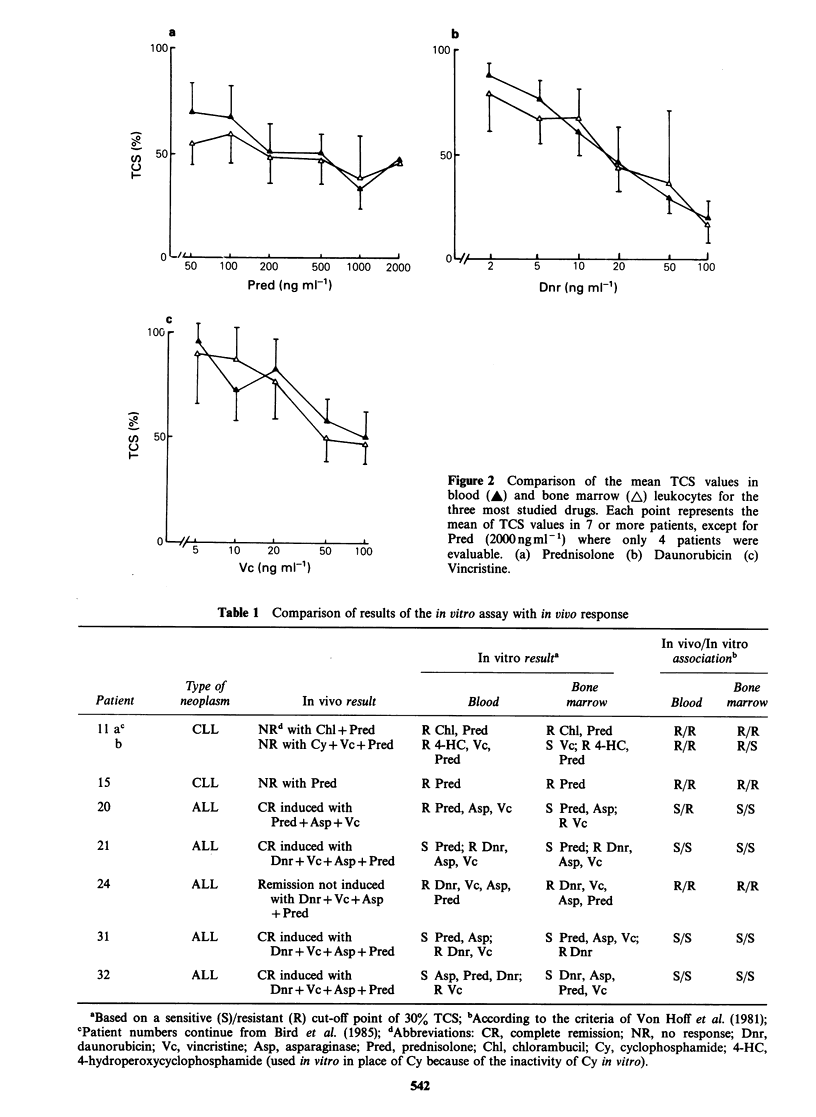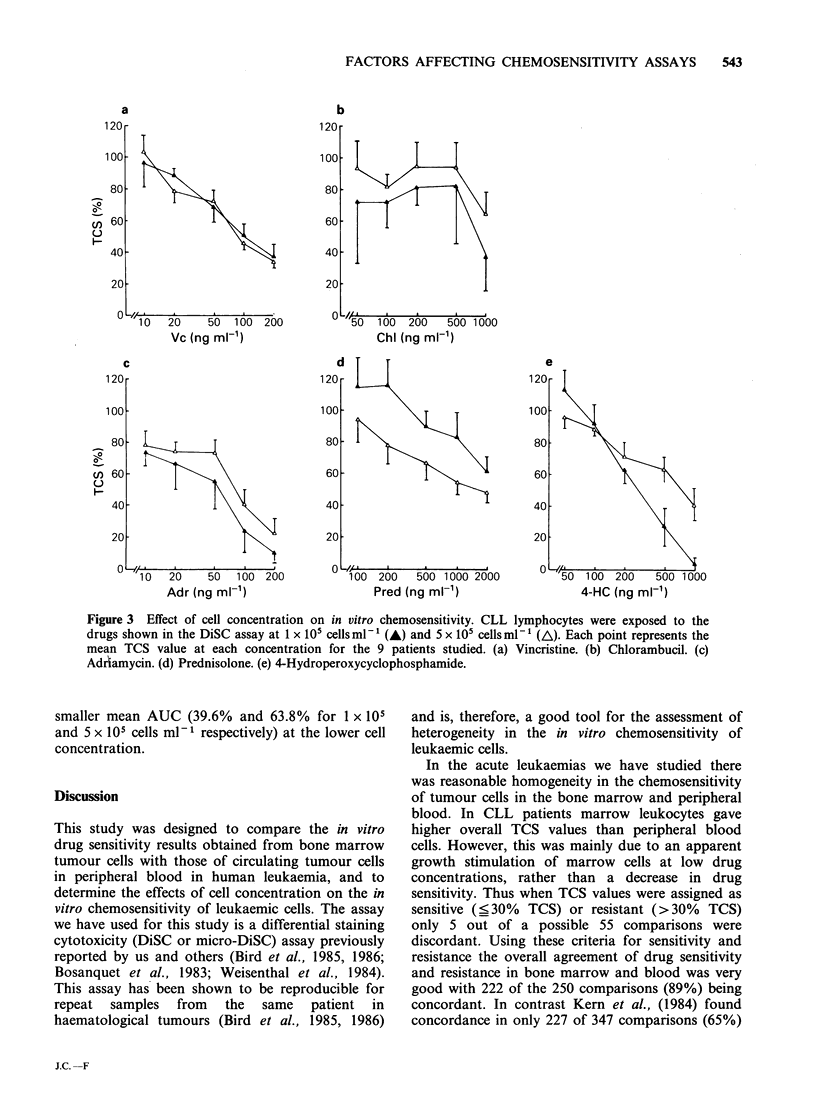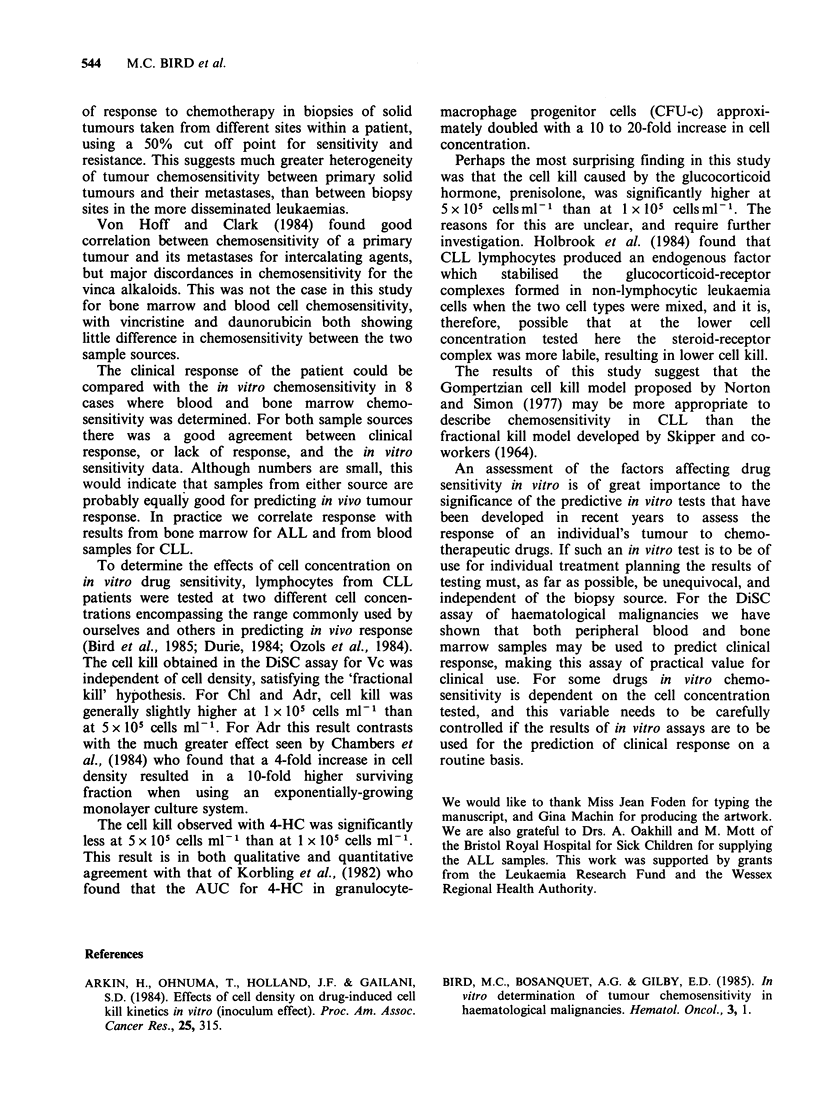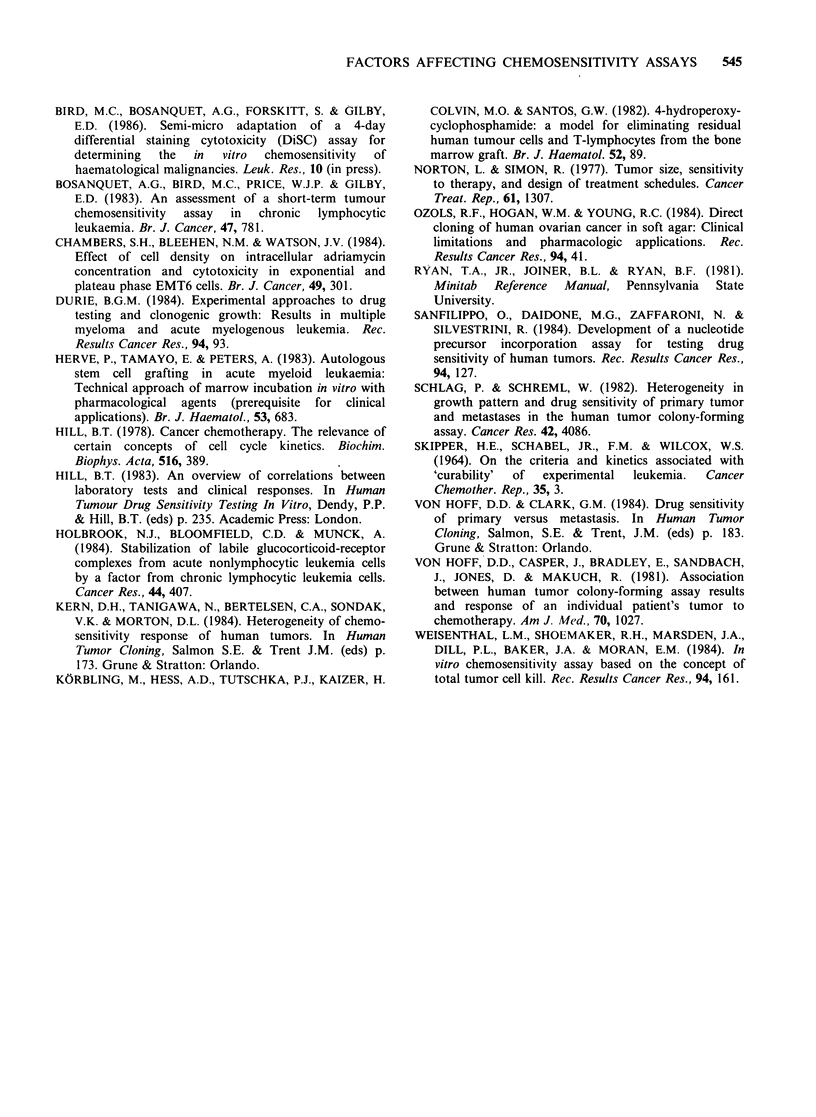# The influence of sample source and cell concentration on the in vitro chemosensitivity of haematological tumours.

**DOI:** 10.1038/bjc.1986.84

**Published:** 1986-04

**Authors:** M. C. Bird, S. Forskitt, E. D. Gilby, A. G. Bosanquet

## Abstract

The Differential Staining Cytotoxicity (DiSC) assay has been used to study the effects of sample source and cell concentration on the in vitro chemosensitivity of haematological malignancies. The chemosensitivity of blood and bone marrow samples was significantly associated (P less than 0.001) in 12 cases where both were tested simultaneously. In 8 of the cases, where the in vitro result could be compared with clinical response, the in vitro and in vivo chemosensitivity was in agreement in 7, for both blood and bone marrow samples. The in vitro chemosensitivity of chronic lymphocytic leukaemia blood lymphocytes was dependent on the cell concentration for 4 out of 5 drugs tested. A five fold reduction in cell number resulted in a significantly greater cell kill with 4-hydroperoxycyclophosphamide, a greater cell kill (not significant) with chlorambucil and adriamycin, and a significantly lower cell kill with prednisolone. The cell concentration did not affect vincristine cytotoxicity. These results suggest that sample source is not an important consideration for the in vitro chemosensitivity of leukaemias, but that the cell concentration tested should not be varied from assay to assay if the results are to be used for comparative purposes.


*Br. J. Cancer* (1986), **53**, 539–545

### The influence of sample source and cell concentration on the *in vitro* chemosensitivity of haematological tumours

M.C. Bird[1]*, S. Forskitt[1], E.D. Gilby[2] & A.G. Bosanquet[1]

*Departments of [1]Clinical Investigation and [2]Medical Oncology, Royal United Hospital, Combe Park, Bath BA1 3NG, UK.*

**Summary** The Differential Staining Cytotoxicity (DiSC) assay has been used to study the effects of sample source and cell concentration on the *in vitro* chemosensitivity of haematological malignancies.

The chemosensitivity of blood and bone marrow samples was significantly associated ($P<0.001$) in 12 cases where both were tested simultaneously. In 8 of the cases, where the *in vitro* result could be compared with clinical response, the *in vitro* and *in vivo* chemosensitivity was in agreement in 7, for both blood and bone marrow samples.

The *in vitro* chemosensitivity of chronic lymphocytic leukaemia blood lymphocytes was dependent on the cell concentration for 4 out of 5 drugs tested. A five fold reduction in cell number resulted in a significantly greater cell kill with 4-hydroperoxycyclophosphamide, a greater cell kill (not significant) with chlorambucil and adriamycin, and a significantly lower cell kill with prednisolone. The cell concentration did not affect vincristine cytotoxicity.

These results suggest that sample source is not an important consideration for the *in vitro* chemosensitivity of leukaemias, but that the cell concentration tested should not be varied from assay to assay if the results are to be used for comparative purposes.

*In vitro* chemosensitivity tests have proved to be of use for predicting tumour response *in vivo* (Hill, 1983). However, there are always a small proportion of discrepancies between the *in vitro* and *in vivo* results. Besides pharmacokinetic differences between the *in vitro* and *in vivo* situations, these discrepancies may be due to heterogeneous chemosensitivity of tumour cells in different sites within one particular tumour-bearing individual, so that sampling from one site may not reflect the overall clinical response of the patient. Such heterogeneity has been shown for solid tumours, both within a primary tumour, and between a primary tumour and its metastases (Schlag & Schreml, 1982; Kern *et al.*, 1984; San Filippo *et al.*, 1984; Von Hoff & Clark, 1984). The chemosensitivity of tumour cells taken from different biopsy sites in haematological tumours has, however, not been similarly investigated. We have, therefore, studied the chemosensitivity of leukocytes taken simultaneously from peripheral blood and bone marrow of patients with chronic lymphocytic leukaemia (CLL), acute lymphoblastic leukaemia (ALL) and acute myeloid leukaemia (AML), using a short-term differential staining cytotoxicity (DiSC) assay previously reported by us and others (Bird *et al.*, 1985, 1986; Bosanquet *et al.*, 1983; Weisenthal *et al.*, 1984).

*Present address: Department of Pathology, Smith Kline and French Research Ltd., The Frythe, Welwyn AL6 9AR, Herts., UK.

Correspondence: A.G. Bosanquet.

Received 7 October 1985; and in revised form, 13 December 1985.

It has been postulated that cell destruction by anticancer drugs follow 'first order kinetics' (Hill, 1978), and that a given treatment destroys a constant fraction of cells, not a fixed number. It follows from this 'fractional kill' hypothesis that cell kill should be independent of cell number for any given concentration of drug. Although this hypothesis holds good for certain exponentially-growing experimental tumours (such as the L1210 leukaemia), it is less satisfactory for most experimental and clinical tumours which seem to more closely follow Gompertzian growth kinetics (Norton & Simon, 1977). Thus there have been reports that, for some agents, cytotoxicity *in vitro* is profoundly affected by the ratio of cell concentration to drug dose, both in established cell lines (Chambers *et al.*, 1984; Arkin *et al.*, 1984) and in primary cultures (Körbling *et al.*, 1982; Hervé *et al.*, 1983). For this reason we have also examined the relationship between cell concentration and cell kill in the DiSC assay for five different drugs, using peripheral blood lymphocytes from CLL patients.

### Materials and methods

### *Samples and cell separation*

For comparison of blood and bone marrow leukocyte chemosensitivity, a bone marrow aspirate and a peripheral blood sample were collected from 12 patients and processed simultaneously. Six patients had ALL, 3 had AML and 3 had CLL. All

© The Macmillan Press Ltd., 1986

the samples were taken at diagnosis (therefore no previous chemotherapy) except for one patient with CLL (patient 6 in Table I). This person had been diagnosed 6 years earlier and received chemotherapy, although in the 6 months prior to the samples being taken he had only received one 4-day course of chlorambucil and prednisolone. Although more pairs of samples were collected, only those showing $>10\%$ tumour cells in both samples could be analysed by the DiSC assay.

For the cell concentration experiments, blood samples were collected from 9 CLL patients who, if they had had chemotherapy, had not received it in the previous three weeks.

Peripheral blood samples (5–10 ml) were collected into potassium EDTA tubes and bone marrow (0.2–1.0 ml) into lithium heparin tubes. Samples were diluted 1:1 (v/v) with PBS, and the leukocyte population (always $\geq 98\%$ viable) was obtained by centrifugation over Ficoll-hypaque as described previously (Bird *et al.*, 1985).

*DiSC assay*

Drugs were made up and stored in the most appropriate media as described elsewhere (Bird *et al.*, 1986). The DiSC or micro-DiSC assay was performed as previously described (Bird *et al.*, 1985, 1986). Briefly, drugs were diluted to the required concentration range with PBS, and incubated with cells ($1 \times 10^5$ ml$^{-1}$ or $5 \times 10^5$ ml$^{-1}$ in RPMI 1640 medium containing 10% foetal calf serum and 24 mM $NaHCO_3$ (RPMI-FCS)) under 5% $CO_2$ at 37°C for 4 days. Single tubes were set up for each drug concentration and six control tubes received PBS in place of the drug. (With this system, the standard deviation on the results has been variously estimated at 12% and 20% (Bird *et al.*, 1985, 1986)). Following this, cells were stained with nigrosin–fast green stain, containing a known number of permanently fixed duck red blood cells (DRBC), and cytocentrifuged onto collagen-coated slides. The slides were then fixed with methanol, and counterstained with a Romanowsky stain. For quantification of results, the ratio of live tumour cells over simultaneously counted DRBC was determined for each slide and the ratio in drug-treated samples expressed as a percentage of that in the control. This expression was termed the tumour cell survival (TCS).

*Statistical analysis*

Paired *t*-tests and calculation of correlation coefficients were performed by using the statistical package, Minitab (Ryan *et al.*, 1981), on a Honeywell Series 60 mainframe computer.

### Results

*Blood and bone marrow chemosensitivity*

Leukocytes from 12 pairs of bone marrow aspirates and peripheral blood samples (6 ALL, 3 AML and 3 CLL) obtained simultaneously were assayed by the DiSC or micro-DiSC assay. The control viability of blood samples at the end of the 4-day assay was significantly higher than those of marrow leukocytes ($P<0.05$; paired *t*-test) with means of 55% and 45% for the two sample sources over the 12 patients studied. A total of 250 drug comparisons were possible for the 12 patients. The TCS values obtained in the assay for the two sample sources were plotted against each other and the results are shown in Figure 1. Overall the data was very well correlated ($r=0.69$; $P \ll 0.001$) and the best fit line (shown in Figure 1*a*) lay close to $x=y$. This relationship remained when the TCS values for ALL and AML patients were plotted separately (Figure 1*b* and *c*). For the 3 CLL patients the data was also well correlated ($P \ll 0.001$) around the best fit line (Figure 1*d*), but there was significant deviation from $x=y$, with the TCS values for marrow being generally higher than those for blood leykocytes (paired *t* test; $P<0.01$).

For further analysis all the TCS values in Figure 1 were assigned as being sensitive ($\leq 30\%$ TCS) or resistant ($>30\%$ TCS). Differences in sensitivity of blood and bone marrow by these criteria were found in 28 of the 250 possible comparisons (11%), comprising 5 (9%) of the CLL comparisons, 3 (6%) of the AML comparisons, and 20 (16%) of the ALL comparisons.

To determine if the category of antineoplastic agent made any difference in the correlation between sensitivity or resistance in blood and bone marrow cells, the mean dose response curves for the three most studied drugs (prednisolone (Pred), daunorubicin (Dnr) and vincristine (Vc)) in blood and bone marrow samples were plotted (Figure 2). The differences between the TCS values were far from significant for the two sample sources (paired *t*-test; $P>0.2$, 0.6 and 0.7 for Pred, Vc and Dnr respectively) and the overall shape of the curves were very similar with the area under the dose-response curve (AUC) for each of the mean curves (shown in Figure 2) being in good agreement. In 8 cases where the *in vitro* results could be compared with clinical response, blood and bone marrow samples were equally accurate in predicting response *in vivo*, giving one false correlation each (Table I).

*Effect of cell concentration on chemosensitivity*

To determine the effects of cell concentration on *in vitro* drug sensitivity, leukocytes from 9 CLL

**Figure 1** Comparison of *in vitro* drug sensitivity in blood and bone marrow leukocytes. Each point represents a paired TCS value. The line shown is the line of best fit. (a) Total data, $r=0.69$ (b) ALL, $r=0.72$ (c) AML, $r=0.80$ (d) CLL, $r=0.53$.

patients were tested at two different cell concentrations ($1\times10^5$ and $5\times10^5$ viable cells $ml^{-1}$) for sensitivity to Vc, Pred, 4-hydroperoxy-cyclophosphamide (4-HC), adriamycin (Adr) and chlorambucil (Chl) in the DiSC assay. The control viability of the cells in the assay at 4 days was significantly greater at the higher seeding density (paired *t*-test, $P<0.001$; mean viability 69% and 46% for $5\times10^5$ and $1\times10^5$ cells $ml^{-1}$ respectively). The TCS values obtained at the two cell concentrations were not significantly different for Vc (paired *t*-test, $P>0.6$). For Chl and Adr the differences in cytotoxicity were not great (paired *t*-test, $0.05<P<0.1$). This is reflected in quite similar mean dose response curves for these three drugs in the 9 patients studied (Figure 3*a,b,c*). TCS values for Pred were significantly higher at $1\times10^5$ cells $ml^{-1}$ than at $5\times10^5$ $ml^{-1}$, for all concentrations of drug tested (paired *t*-test $P<0.01$). The overall shape of the mean dose response curves was similar at both cell concentrations (Figure 3*d*). For 4-HC the TCS values were significantly lower at $1\times10^5$ cells $ml^{-1}$ than at $5\times10^5$ $ml^{-1}$ (overall $P<0.05$) with the greatest differences being at the higher drug concentrations tested (mean differences 3% at 100 ng $ml^{-1}$ but 43% at 1000 ng $ml^{-1}$). The mean dose response curve for the 9 patients was steeper for $1\times10^5$ cells $ml^{-1}$ (Figure 3*e*), resulting in a

**Figure 2** Comparison of the mean TCS values in blood (▲) and bone marrow (△) leukocytes for the three most studied drugs. Each point represents the mean of TCS values in 7 or more patients, except for Pred ($2000 ng ml^{-1}$) where only 4 patients were evaluable. (a) Prednisolone (b) Daunorubicin (c) Vincristine.

**Table 1** Comparison of results of the *in vitro* assay with *in vivo* response

| *Patient* | *Type of neoplasm* | In vivo *result* | In vitro *result*[a] | | In vivo/In vitro *association*[b] | |
|---|---|---|---|---|---|---|
| | | | *Blood* | *Bone marrow* | *Blood* | *Bone marrow* |
| 11 a[c] | CLL | NR[d] with Chl + Pred | R Chl, Pred | R Chl, Pred | R/R | R/R |
| b | | NR with Cy + Vc + Pred | R 4-HC, Vc, Pred | S Vc; R 4-HC, Pred | R/R | R/S |
| 15 | CLL | NR with Pred | R Pred | R Pred | R/R | R/R |
| 20 | ALL | CR induced with Pred + Asp + Vc | R Pred, Asp, Vc | S Pred, Asp; R Vc | S/R | S/S |
| 21 | ALL | CR induced with Dnr + Vc + Asp + Pred | S Pred; R Dnr, Asp, Vc | S Pred; R Dnr, Asp, Vc | S/S | S/S |
| 24 | ALL | Remission not induced with Dnr + Vc + Asp + Pred | R Dnr, Vc, Asp, Pred | R Dnr, Vc, Asp, Pred | R/R | R/R |
| 31 | ALL | CR induced with Dnr + Vc + Asp + Pred | S Pred, Asp; R Dnr, Vc | S Pred, Asp, Vc; R Dnr | S/S | S/S |
| 32 | ALL | CR induced with Dnr + Vc + Asp + Pred | S Asp, Pred, Dnr; R Vc | S Dnr, Asp, Pred, Vc | S/S | S/S |

[a]Based on a sensitive (S)/resistant (R) cut-off point of 30% TCS; [b]According to the criteria of Von Hoff *et al.* (1981); [c]Patient numbers continue from Bird *et al.* (1985); [d]Abbreviations: CR, complete remission; NR, no response; Dnr, daunorubicin; Vc, vincristine; Asp, asparaginase; Pred, prednisolone; Chl, chlorambucil; Cy, cyclophosphamide; 4-HC, 4-hydroperoxycyclophosphamide (used *in vitro* in place of Cy because of the inactivity of Cy *in vitro*).

**Figure 3** Effect of cell concentration on *in vitro* chemosensitivity. CLL lymphocytes were exposed to the drugs shown in the DiSC assay at $1 \times 10^5$ cells ml$^{-1}$ (▲) and $5 \times 10^5$ cells ml$^{-1}$ (△). Each point represents the mean TCS value at each concentration for the 9 patients studied. (a) Vincristine. (b) Chlorambucil. (c) Adriamycin. (d) Prednisolone. (e) 4-Hydroperoxycyclophosphamide.

smaller mean AUC (39.6% and 63.8% for $1 \times 10^5$ and $5 \times 10^5$ cells ml$^{-1}$ respectively) at the lower cell concentration.

### Discussion

This study was designed to compare the *in vitro* drug sensitivity results obtained from bone marrow tumour cells with those of circulating tumour cells in peripheral blood in human leukaemia, and to determine the effects of cell concentration on the *in vitro* chemosensitivity of leukaemic cells. The assay we have used for this study is a differential staining cytotoxicity (DiSC or micro-DiSC) assay previously reported by us and others (Bird *et al.*, 1985, 1986; Bosanquet *et al.*, 1983; Weisenthal *et al.*, 1984). This assay has been shown to be reproducible for repeat samples from the same patient in haematological tumours (Bird *et al.*, 1985, 1986) and is, therefore, a good tool for the assessment of heterogeneity in the *in vitro* chemosensitivity of leukaemic cells.

In the acute leukaemias we have studied there was reasonable homogeneity in the chemosensitivity of tumour cells in the bone marrow and peripheral blood. In CLL patients marrow leukocytes gave higher overall TCS values than peripheral blood cells. However, this was mainly due to an apparent growth stimulation of marrow cells at low drug concentrations, rather than a decrease in drug sensitivity. Thus when TCS values were assigned as sensitive ($\leq 30\%$ TCS) or resistant ($>30\%$ TCS) only 5 out of a possible 55 comparisons were discordant. Using these criteria for sensitivity and resistance the overall agreement of drug sensitivity and resistance in bone marrow and blood was very good with 222 of the 250 comparisons (89%) being concordant. In contrast Kern *et al.*, (1984) found concordance in only 227 of 347 comparisons (65%)

of response to chemotherapy in biopsies of solid tumours taken from different sites within a patient, using a 50% cut off point for sensitivity and resistance. This suggests much greater heterogeneity of tumour chemosensitivity between primary solid tumours and their metastases, than between biopsy sites in the more disseminated leukaemias.

Von Hoff and Clark (1984) found good correlation between chemosensitivity of a primary tumour and its metastases for intercalating agents, but major discordances in chemosensitivity for the vinca alkaloids. This was not the case in this study for bone marrow and blood cell chemosensitivity, with vincristine and daunorubicin both showing little difference in chemosensitivity between the two sample sources.

The clinical response of the patient could be compared with the *in vitro* chemosensitivity in 8 cases where blood and bone marrow chemosensitivity was determined. For both sample sources there was a good agreement between clinical response, or lack of response, and the *in vitro* sensitivity data. Although numbers are small, this would indicate that samples from either source are probably equally good for predicting *in vivo* tumour response. In practice we correlate response with results from bone marrow for ALL and from blood samples for CLL.

To determine the effects of cell concentration on *in vitro* drug sensitivity, lymphocytes from CLL patients were tested at two different cell concentrations encompassing the range commonly used by ourselves and others in predicting *in vivo* response (Bird *et al.*, 1985; Durie, 1984; Ozols *et al.*, 1984). The cell kill obtained in the DiSC assay for Vc was independent of cell density, satisfying the 'fractional kill' hypothesis. For Chl and Adr, cell kill was generally slightly higher at $1 \times 10^5$ cells $ml^{-1}$ than at $5 \times 10^5$ cells $ml^{-1}$. For Adr this result contrasts with the much greater effect seen by Chambers *et al.*, (1984) who found that a 4-fold increase in cell density resulted in a 10-fold higher surviving fraction when using an exponentially-growing monolayer culture system.

The cell kill observed with 4-HC was significantly less at $5 \times 10^5$ cells $ml^{-1}$ than at $1 \times 10^5$ cells $ml^{-1}$. This result is in both qualitative and quantitative agreement with that of Korbling *et al.*, (1982) who found that the AUC for 4-HC in granulocyte-macrophage progenitor cells (CFU-c) approximately doubled with a 10 to 20-fold increase in cell concentration.

Perhaps the most surprising finding in this study was that the cell kill caused by the glucocorticoid hormone, prenisolone, was significantly higher at $5 \times 10^5$ cells $ml^{-1}$ than at $1 \times 10^5$ cells $ml^{-1}$. The reasons for this are unclear, and require further investigation. Holbrook *et al.* (1984) found that CLL lymphocytes produced an endogenous factor which stabilised the glucocorticoid-receptor complexes formed in non-lymphocytic leukaemia cells when the two cell types were mixed, and it is, therefore, possible that at the lower cell concentration tested here the steroid-receptor complex was more labile, resulting in lower cell kill.

The results of this study suggest that the Gompertzian cell kill model proposed by Norton and Simon (1977) may be more appropriate to describe chemosensitivity in CLL than the fractional kill model developed by Skipper and co-workers (1964).

An assessment of the factors affecting drug sensitivity *in vitro* is of great importance to the significance of the predictive *in vitro* tests that have been developed in recent years to assess the response of an individual's tumour to chemotherapeutic drugs. If such an *in vitro* test is to be of use for individual treatment planning the results of testing must, as far as possible, be unequivocal, and independent of the biopsy source. For the DiSC assay of haematological malignancies we have shown that both peripheral blood and bone marrow samples may be used to predict clinical response, making this assay of practical value for clinical use. For some drugs *in vitro* chemosensitivity is dependent on the cell concentration tested, and this variable needs to be carefully controlled if the results of *in vitro* assays are to be used for the prediction of clinical response on a routine basis.

We would like to thank Miss Jean Foden for typing the manuscript, and Gina Machin for producing the artwork. We are also grateful to Drs. A. Oakhill and M. Mott of the Bristol Royal Hospital for Sick Children for supplying the ALL samples. This work was supported by grants from the Leukaemia Research Fund and the Wessex Regional Health Authority.

### References

ARKIN, H., OHNUMA, T., HOLLAND, J.F. & GAILANI, S.D. (1984). Effects of cell density on drug-induced cell kill kinetics *in vitro* (inoculum effect). *Proc. Am. Assoc. Cancer Res.*, **25**, 315.

BIRD, M.C., BOSANQUET, A.G. & GILBY, E.D. (1985). *In vitro* determination of tumour chemosensitivity in haematological malignancies. *Hematol. Oncol.*, **3**, 1.

BIRD, M.C., BOSANQUET, A.G., FORSKITT, S. & GILBY, E.D. (1986). Semi-micro adaptation of a 4-day differential staining cytotoxicity (DiSC) assay for determining the *in vitro* chemosensitivity of haematological malignancies. *Leuk. Res.*, **10** (in press).

BOSANQUET, A.G., BIRD, M.C., PRICE, W.J.P. & GILBY, E.D. (1983). An assessment of a short-term tumour chemosensitivity assay in chronic lymphocytic leukaemia. *Br. J. Cancer*, **47**, 781.

CHAMBERS, S.H., BLEEHEN, N.M. & WATSON, J.V. (1984). Effect of cell density on intracellular adriamycin concentration and cytotoxicity in exponential and plateau phase EMT6 cells. *Br. J. Cancer*, **49**, 301.

DURIE, B.G.M. (1984). Experimental approaches to drug testing and clonogenic growth: Results in multiple myeloma and acute myelogenous leukemia. *Rec. Results Cancer Res.*, **94**, 93.

HERVE, P., TAMAYO, E. & PETERS, A. (1983). Autologous stem cell grafting in acute myeloid leukaemia: Technical approach of marrow incubation *in vitro* with pharmacological agents (prerequisite for clinical applications). *Br. J. Haematol.*, **53**, 683.

HILL, B.T. (1978). Cancer chemotherapy. The relevance of certain concepts of cell cycle kinetics. *Biochim. Biophys. Acta*, **516**, 389.

HILL, B.T. (1983). An overview of correlations between laboratory tests and clinical responses. In *Human Tumour Drug Sensitivity Testing In Vitro*, Dendy, P.P. & Hill, B.T. (eds) p. 235. Academic Press: London.

HOLBROOK, N.J., BLOOMFIELD, C.D. & MUNCK, A. (1984). Stabilization of labile glucocorticoid-receptor complexes from acute nonlymphocytic leukemia cells by a factor from chronic lymphocytic leukemia cells. *Cancer Res.*, **44**, 407.

KERN, D.H., TANIGAWA, N., BERTELSEN, C.A., SONDAK, V.K. & MORTON, D.L. (1984). Heterogeneity of chemosensitivity response of human tumors. In *Human Tumor Cloning*, Salmon S.E. & Trent J.M. (eds) p. 173. Grune & Stratton: Orlando.

KÖRBLING, M., HESS, A.D., TUTSCHKA, P.J., KAIZER, H. COLVIN, M.O. & SANTOS, G.W. (1982). 4-hydroperoxy-cyclophosphamide: a model for eliminating residual human tumour cells and T-lymphocytes from the bone marrow graft. *Br. J. Haematol.* **52**, 89.

NORTON, L. & SIMON, R. (1977). Tumor size, sensitivity to therapy, and design of treatment schedules. *Cancer Treat. Rep.*, **61**, 1307.

OZOLS, R.F., HOGAN, W.M. & YOUNG, R.C. (1984). Direct cloning of human ovarian cancer in soft agar: Clinical limitations and pharmacologic applications. *Rec. Results Cancer Res.*, **94**, 41.

RYAN, T.A., JR., JOINER, B.L. & RYAN, B.F. (1981). *Minitab Reference Manual,* Pennsylvania State University.

SANFILIPPO, O., DAIDONE, M.G., ZAFFARONI, N. & SILVESTRINI, R. (1984). Development of a nucleotide precursor incorporation assay for testing drug sensitivity of human tumors. *Rec. Results Cancer Res.*, **94**, 127.

SCHLAG, P. & SCHREML, W. (1982). Heterogeneity in growth pattern and drug sensitivity of primary tumor and metastases in the human tumor colony-forming assay. *Cancer Res.* **42**, 4086.

SKIPPER, H.E., SCHABEL, JR., F.M. & WILCOX, W.S. (1964). On the criteria and kinetics associated with 'curability' of experimental leukemia. *Cancer Chemother. Rep.*, **35**, 3.

VON HOFF, D.D. & CLARK, G.M. (1984). Drug sensitivity of primary versus metastasis. In *Human Tumor Cloning,* Salmon, S.E. & Trent, J.M. (eds) p. 183. Grune & Stratton: Orlando.

VON HOFF, D.D., CASPER, J., BRADLEY, E., SANDBACH, J., JONES, D. & MAKUCH, R. (1981). Association between human tumor colony-forming assay results and response of an individual patient's tumor to chemotherapy. *Am J. Med.*, **70**, 1027.

WEISENTHAL, L.M., SHOEMAKER, R.H., MARSDEN, J.A., DILL, P.L., BAKER, J.A. & MORAN, E.M. (1984). *In vitro* chemosensitivity assay based on the concept of total tumor cell kill. *Rec. Results Cancer Res.*, **94**, 161.